# Human Serum PCSK9 Is Elevated at Parturition in Comparison to Nonpregnant Subjects While Serum PCSK9 from Umbilical Cord Blood is Lower Compared to Maternal Blood

**DOI:** 10.1155/2013/341632

**Published:** 2013-06-05

**Authors:** Patricia Peticca, Angela Raymond, Andrée Gruslin, Marion Cousins, Ejibunmi Adetola, Hussein Abujrad, Janice Mayne, Teik Chye Ooi

**Affiliations:** ^1^Clinical Research Laboratory, Division of Endocrinology and Metabolism, Department of Medicine, The Ottawa Hospital, Riverside Campus, University of Ottawa, 1967 Riverside Drive, Ottawa, ON, Canada K1H 7W9; ^2^Ottawa Institute of Systems Biology, Department of Biochemistry, Microbiology and Immunology, University of Ottawa, 451 Smyth Road, Ottawa, ON, Canada K1H 8M5; ^3^Division of Maternal Fetal Medicine, Departments of Obstetrics and Gynaecology and Cellular Molecular Medicine, University of Ottawa, 501 Smyth Road, Box 804, Ottawa, ON, Canada K1H 8L6; ^4^Chronic Disease Program, Ottawa Hospital Research Institute, The Ottawa Hospital, 501 Smyth Road, Ottawa, ON, Canada K1H 8L6; ^5^Department of Obstetrics and Gynaecology, Winchester District Memorial Hospital, 566 Louise Street, Winchester, ON, Canada K0C 2K0

## Abstract

*Background*. Serum lipids including total cholesterol (TC), triglycerides (TG), and low density lipoprotein cholesterol (LDL-C) are increased in pregnancy. Serum proprotein convertase subtilisin kexin 9 (PCSK9) is a significant player in lipoprotein metabolism. Circulating PCSK9 downregulates the LDL receptor on the surface of the liver, inhibiting clearance of LDL-C. Therefore, our study assessed serum PCSK9 concentrations at parturition (Maternal) compared to a nonpregnant (Control) cohort, as well as between mother and newborn (Maternal and Newborn). *Methods*. Blood was collected from women at parturition and from umbilical cords. Serum lipids and PCSK9 were measured and data were analysed for significance by Mann-Whitney *U* test at *P* < 0.05 and presented as median levels. Spearman's correlations were made at a 95% confidence interval. *Results*. Serum PCSK9 was significantly higher in Maternal versus Control cohorts (493.1 versus 289.7 ng/mL; *P* < 0.001, resp.), while the Newborn cohort was significantly lower than Maternal (278.2 versus 493.1 ng/mL; *P* < 0.0001, resp.). PCSK9 was significantly correlated with TC and HDL-C in Maternal and with TC, LDL-C, and HDL-C in Newborn cohorts. *Conclusions*. Our study provides the first quantitative report on PCSK9 in pregnancy (at parturition) and in umbilical cord blood. Further research will determine how these changes may affect lipoprotein levels during this physiological state.

## 1. Introduction

Pregnancy is associated with steady increases in maternal serum concentrations of total cholesterol (TC) and low density lipoprotein cholesterol (LDL-C) from first to third trimester [1, 2]. Other changes include an increase in high density lipoprotein cholesterol (HDL-C), triglycerides (TG), serum apolipoprotein B (apoB), and apolipoprotein AI (apoAI) levels and a net increase in the apoB/apoAI ratio [1, 2]. In contrast, umbilical cord blood has lower lipid levels than adults, including TG, LDL-C, and HDL-C subfractions [3]. In addition, HDL is the major cholesterol-containing particle in cord blood, unlike adult blood where it is LDL [3]. The molecular mechanisms involving lipoprotein changes during pregnancy and their influence on cord blood levels have yet to be fully elucidated [3].

Proprotein convertase subtilisin kexin 9 (PCSK9), a secreted glycoprotein and member of the proprotein convertase family of mammalian serine proteases, has emerged as a significant player in lipoprotein metabolism since its discovery in 2003 [4]. Population studies have shown that PCSK9 gain of function variants associate with high LDL-C levels and autosomal dominant hypercholesterolemia [5, 6], whereas loss of function variants associate with low LDL-C levels [7, 8]. In addition, there is a positive correlation between serum PCSK9 and LDL-C levels [9–12], which is not surprising given that secreted PCSK9, acting as an escort protein, binds to low density lipoprotein receptors (LDLR) on the cell surface of the liver, thereafter interfering with its natural course of recycling [13, 14] and routing the LDLR to the lysosome for degradation [15, 16], resulting in accumulation of LDL particles in circulation. Alterations in serum PCSK9 levels and- or its positive correlation to LDL-C have been documented in several physiological and pathological states in which lipid levels are also affected: circulating PCSK9 follows a diurnal rhythm with cholesterol synthesis [17] and is decreased during fasting [18, 19], and PCSK9 is upregulated by the commonly prescribed lipid-lowering statins thereby blunting their effect [20–22], but PCSK9 is not correlated with LDL-C in controlled Type II diabetes and is, in fact, negatively correlated in uncontrolled Type II diabetes [23]. Recently, placental PCSK9 protein levels were decreased in both normal weight and obese mothers with gestational diabetes mellitus when compared to normal weight nondiabetic mothers [24]. This occurred without changes in placental PCSK9 mRNA [24]. However, to our knowledge, there are no published data on serum PCSK9 levels in pregnancy. Based upon PCSK9 correlations with lipids in other studies, we hypothesized that serum PCSK9 levels would be elevated in the hyperlipidemic stage of pregnancy. Our study demonstrates for the first time that PCSK9 concentrations are indeed higher at parturition than in nonpregnancy. We also demonstrate that umbilical cord blood levels of PCSK9 are significantly lower than maternal blood levels.

## 2. Materials and Methods

### 2.1. Study Groups

#### 2.1.1. Maternal and Newborn Cohorts

 Study protocols were approved by The Ottawa Hospital Research Ethics Board. All subjects provided informed written consent prior to the collection of samples. Maternal and umbilical cord blood samples were obtained post hoc from a study of pregnant women at parturition (*n* = 41), who delivered at The Ottawa Hospital between 2007 and 2009 and included women with uncomplicated pregnancy (Normal; *n* = 15) and those with a diagnosis of preeclampsia/toxemia (PET; *n* = 15) or intrauterine growth restriction (IUGR; *n* = 11). Figure S1, in Supplementary Material available online at http://dx.doi.org/10.1155/2013/341632, shows the scatter plot distributions of serum PCSK9 and lipids for the Maternal subgroups (for both mother and newborn) in comparison to the overall Maternal and Newborn distributions; however no subgroup comparisons were carried out because subgrouped data were not significantly powered.

#### 2.1.2. Control Cohort

A group of women aged ≤40 comprised our nonpregnant group. These samples were obtained post hoc from a cohort originally recruited to the Foustanellas Endocrine and Diabetes Centre at the Riverside Campus of The Ottawa Hospital, as part of a study on gender-specific differences in PCSK9 [9]. The mean age of our Control cohort was 31.7 years which was similar to that of the Maternal cohort with a mean age of 29.7 years (31.4,   29, and 28.2 years for Normal, PET, and IUGR subgroups, resp.). 

### 2.2. Sample Collection

#### 2.2.1. Maternal and Cord Samples

Nonfasting pregnant subjects had their blood drawn when they presented in active labour. Umbilical cord blood (Newborn cohort) was collected at the time of delivery. Samples were then stored at 4°C and centrifuged within 48 hours, and the sera were stored at −80°C.

#### 2.2.2. Control Samples

Samples were collected as per Mayne et al. (2007) [9]. Overnight fasted blood samples were collected into SST-vacutainer tubes, and sera stored at −80°C.

### 2.3. Measurement of Serum Lipids

TC and TG were measured using enzymatic methods (Diagnostics Chemicals Limited) on a Roche COBAS Mira analyzer with an intra-assay coefficient of variability (CV) of 1.4% and 1.6%, respectively, and an interassay CV of 6.6 mmol/L, 3.8%, 3.2 mmol/L, 3.5%, 2.2 mmol/L, 4.0%, and 0.9 mmol/L, 7.3%, respectively. HDL-C was measured using a direct enzymatic method (Diagnostics Chemicals Limited) on a Roche COBAS Mira analyzer with an intra-assay CV of 1.5% and an inter-assay CV of 1.4 mmol/L, 5.7%. LDL-C was calculated by Friedewald's equation. 

### 2.4. Measurement of Serum PCSK9

Serum PCSK9 was measured using the CircuLex Human PCSK9 ELISA from CycLex Co. (Japan). All samples were quantified 3x with an intra-assay CV of 1.5%–2.6% and an inter-assay CV of 2.9%–7.1%. 

### 2.5. Statistical Analysis

Comparative data are represented as median +/− interquartile range. Power analysis was carried out to ensure that all comparisons were powered at >0.80 and alpha 0.05 using PASS^12^ NCSS Statistical Software. The data were tested for normality using the Kolmogorov-Smirnov test, and the Mann Whitney *U* test for nonparametric data was used to compare groups. All tests were two-sided with significance at *P* < 0.05. Correlations were made using Spearman's correlation test at a 95% confidence interval. GraphPad Prism 5.0 was used for all statistical analyses except where otherwise is noted.

## 3. Results

### 3.1. Serum PCSK9 and Lipids in Control versus Maternal Cohorts

**Figure 1:**
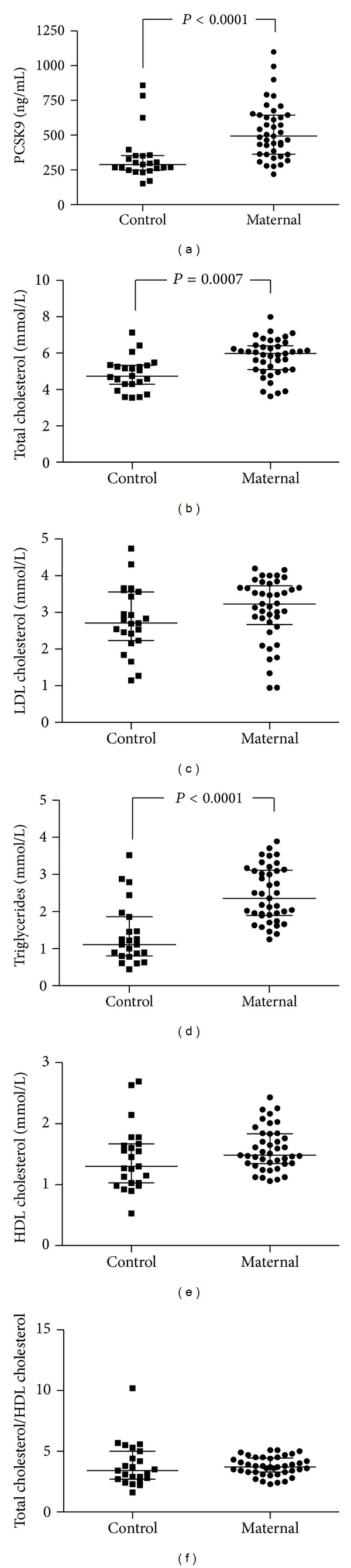
The distribution of serum PCSK9 and lipoproteins in our Control versus Maternal cohorts. Dots represent individual subjects. Bars indicate median ± interquartile range and significance determined by Mann-Whitney *U* at *P* < 0.05.

Figure 1 shows scatter plot distributions of serum PCSK9 and lipids in Control and Maternal cohorts. Serum PCSK9 was significantly elevated in the Maternal cohort compared to the Control cohort by 70.2% (Figure 1(a): 493.1 versus 289.7 ng/mL; *P* < 0.001, resp.). The Maternal cohort showed significantly higher concentrations of TC (26.2%) and TG (112.6%) when compared to the Control cohort (Figure 1(b): 5.98 versus 4.74 mmol/L, *P* = 0.0007 and Figure 1(d): 2.36 versus 1.11 mmol/L, *P* < 0.0001, resp.). No significant difference was observed for serum LDL-C, HDL-C, or TC/HDL-C (Figures 1(c), 1(e), and 1(f), resp.).

### 3.2. Correlation of PCSK9 with Lipids in Control and Maternal Cohorts

**Figure 2:**
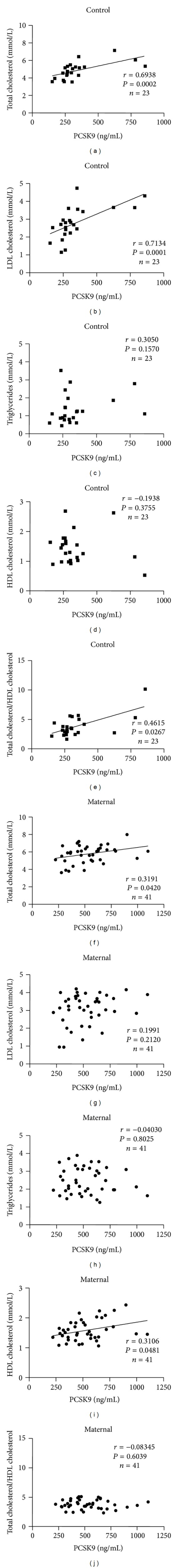
Correlations in Control (A-E) and Maternal (F-J) cohorts between serum PCSK9 (ng/mL) and TC (mmol/L: A and F), LDL-C (mmol/L: B and G), TG (mmol/L: C and H), HDL-C (mmol/L: D and I) and TC/HDL-C (E and J). Correlations were made using the Spearman's correlation test at a 95% confidence interval.

Figure 2 shows the relationship between serum PCSK9 and lipids for both Control and Maternal cohorts by the Spearman analyses. In the Control cohort, serum concentrations of PCSK9 were significantly positively correlated with TC (Figure 2(a): *r* = 0.6938; *P* = 0.0002), LDL-C (Figure 2(b): *r* = 0.7134; *P* = 0.0001), and TC/HDL-C (Figure 2(e): *r* = 0.4615; *P* = 0.0267) but not with TG or HDL-C. Correlations in our Maternal cohort were altered (Figures 2(c), and 2(d), resp.). Maternal PCSK9 was significantly positively associated with TC (Figure 2(f): *r* = 0.3191; *P* = 0.0420) and HDL-C (Figure 2(i): *r* = 0.3106; *P* = 0.0481) but not with LDL-C, TG, or the TC/HDL-C ratio (Figures 2(g), 2(h), and 2(j), resp.). 

### 3.3. Serum PCSK9 and Lipids in Maternal versus Newborn Cohorts

**Figure 3:**
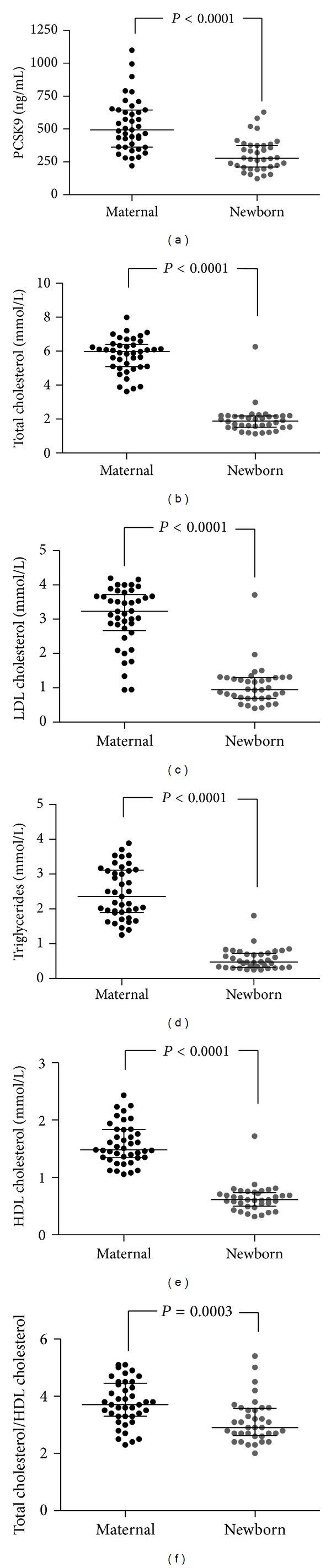
The distribution of serum PCSK9 and lipoproteins in our Maternal versus Newborn cohorts. Dots represent individual subjects. Bars indicate median ± interquartile range and significance determined by Mann-Whitney *U* at *P* < 0.05.

Figure 3 shows that the concentration of serum PCSK9 was 43.6% significantly lower in Newborn when compared to Maternal samples (Figure 3(a): 278.2 versus 493.1 ng/mL; *P* < 0.0001, resp.). Figures 3(b)–3(f) show that TC, LDL-C, TG, HDL-C, and TC/HDL-C are significantly lower in Newborn samples when compared to the Maternal cohort (*P* < 0.0001; *P* < 0.0001; *P* < 0.0001; *P* = 0.0003, resp.). 

### 3.4. Correlation of PCSK9 with Lipids in Newborn Cohorts

**Figure 4:**
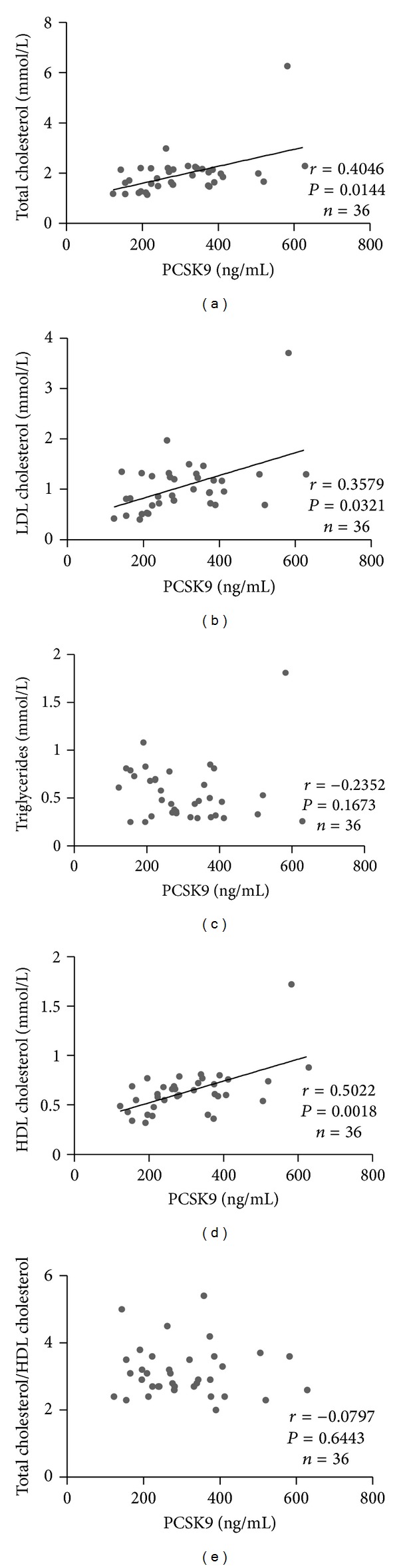
Correlations between serum PCSK9 (ng/mL) and (A) TC (mmol/L), (B) LDLC (mmol/L), (C) TG (mmol/L), (D) HDL-C (mmol/L), and (E) TC/HDL-C for our Newborn cohort. Correlations were made using the Spearman's correlation test at a 95% confidence interval.

Figure 4 shows the relationship between serum PCSK9 and lipids in the Newborn cohort. There were significant positive correlations between serum PCSK9 and TC (Figure 4(a): *r* = 0.4046; *P* = 0.0144), LDL-C (Figure 4(b): *r* = 0.3579; *P* = 0.0321), and HDL-C (Figure 4(d): *r* = 0.5022; *P* = 0.0018) but not TG or TC/HDL-C (Figures 4(c), and 4(e), resp.).

## 4. Discussion

This study provides the first quantitative analysis of maternal serum PCSK9 at parturition and establishes that serum PCSK9 levels were significantly elevated in comparison to Controls. It is worth noting that our Maternal samples were collected in a nonfasting state while Control subjects were fasted overnight. Although serum PCSK9 levels are reportedly downregulated by fasting, it is important to note that it is prolonged fasting of >36 hours that significantly changes circulating PCSK9 levels [17, 19]. Our Control subjects fasted for ≤12 hours, a time interval not reported to significantly affect PCSK9 levels [17, 19]. Additionally, it has been reported that glucose intake has no short-term effect on PCSK9 expression, at both physiological and hyperglycaemic concentrations [18], making it unlikely that higher PCSK9 levels in our nonfasted Maternal subjects were due to glucose intake.

We showed a significant positive correlation of PCSK9 with TC, LDL-C, and TC/HDL-C [9–11] but not with TG or HDL-C in our Control subjects. In fact, correlations with TG and HDL-C are study specific and are not always observed [9, 10, 25]. In our Maternal cohort the relationship between PCSK9 and TC was maintained, while Maternal PCSK9 was also now positively correlated with HDL-C but not with LDL-C nor TC/HDL-C. These observed changes in PCSK9 to lipoprotein relationships, as measured by Spearman's correlation, may be influenced by the many lipid changes, particularly at the point of parturition, that occur during pregnancy [1, 2]. Indeed, at later stages of pregnancy, prior to parturition, LDL-C levels begin to decrease relative to earlier stages in pregnancy [2]. A discordant decrease in LDL-C relative to PCSK9 levels over pregnancy could disrupt the positive association observed in our Control cohort in comparison to our Maternal cohort. A longitudinal study of PCSK9 and lipid levels over pregnancy would establish this effect. 

Our study is also the first to report on Newborn PCSK9 levels as reflected in their umbilical cord blood. It is well known that Newborn lipids are significantly lower than maternal lipid levels [3, 26–28]. Our lipid findings are consistent with those of Bansal et al. (2005) [3] that Newborn TC and LDL-C are approximately one-third the levels found in Maternal blood, whereas HDL-C and TG are reduced by half. Herein, we report the new finding that the Newborn has significantly lower PCSK9 concentrations than Maternal serum (by 43.6%). PCSK9 circulates as a heterodimer of a 63 kDa glycoprotein and a 14 kDa propeptide [4, 29]. We speculate that at this molecular size, circulating PCSK9 is unlikely to be transported from Maternal to fetal circulation [30] and that the concentration of PCSK9 in umbilical cord blood likely reflects synthesis of the protein by the developing fetus. Moreover, PCSK9 was strongly correlated with TC, LDL-C, and HDL-C in Newborn. Little is known about human fetal expression of PCSK9 and the influence of placental function on PCSK9 metabolism or *vice versa*. At later stages of mouse embryonic development however, PCSK9 was expressed in the liver, kidneys, small intestine, and restricted regions of the cerebellum[4]. 

## 5. Conclusion

In summary, this first-ever report of serum PCSK9 in pregnancy provides early observations of a significantly different PCSK9 status at parturition, compared to the nonpregnant state, as well as the first measurement of baseline levels of PCSK9 in the Newborn. The underlying mechanism(s) for serum PCSK9 changes during pregnancy is unknown. However, the possible effect(s) of pregnancy-related hormones, such as estrogens, progesterone, and human placental lactogen, should be considered. These data highlight a need for further research into the expression, regulation, and function of PCSK9 during pregnancy, including a longitudinal cross-sectional study designed from preconception to postpartum. 

## Supplementary Material

Figure S1: Scatter plot distributions of serum PCSK9 and lipids for the Maternal subgroups in comparison to the overall Maternal distribution, and for the Maternal subgroups (in both mother and newborn) to the overall Maternal and Newborn distributions of PCSK9 and lipids. Maternal and Newborn samples were subdivided as Normal, PET (pre-eclampsia) and IUGR (intrauterine growth restriction). Pregnancy was defined as Normal if associated with age appropriate fetal weights, in the absence of maternal disease. PET was defined as having maternal blood pressure of >140/90 and proteinuria >300 mg of protein in a 24 hr urine collection, or 2+ protein on urine dipstick. IUGR was defined as an estimated fetal weight below the 5^th^ percentile for gestational age by ultrasound. Dots represent individual subjects. Bars indicate median ± interquartile range. No subgroup comparisons were carried out because subgrouped data were not significantly powered.
