# Supplementary figures and images for: Human Serum PCSK9 Is Elevated at Parturition in Comparison to Nonpregnant Subjects While Serum PCSK9 from Umbilical Cord Blood is Lower Compared to Maternal Blood
Source: ISRN Endocrinol. 2013 Jun 5;2013:341632. doi: 10.1155/2013/341632 (PMC3687493; doi:10.1155/2013/341632)

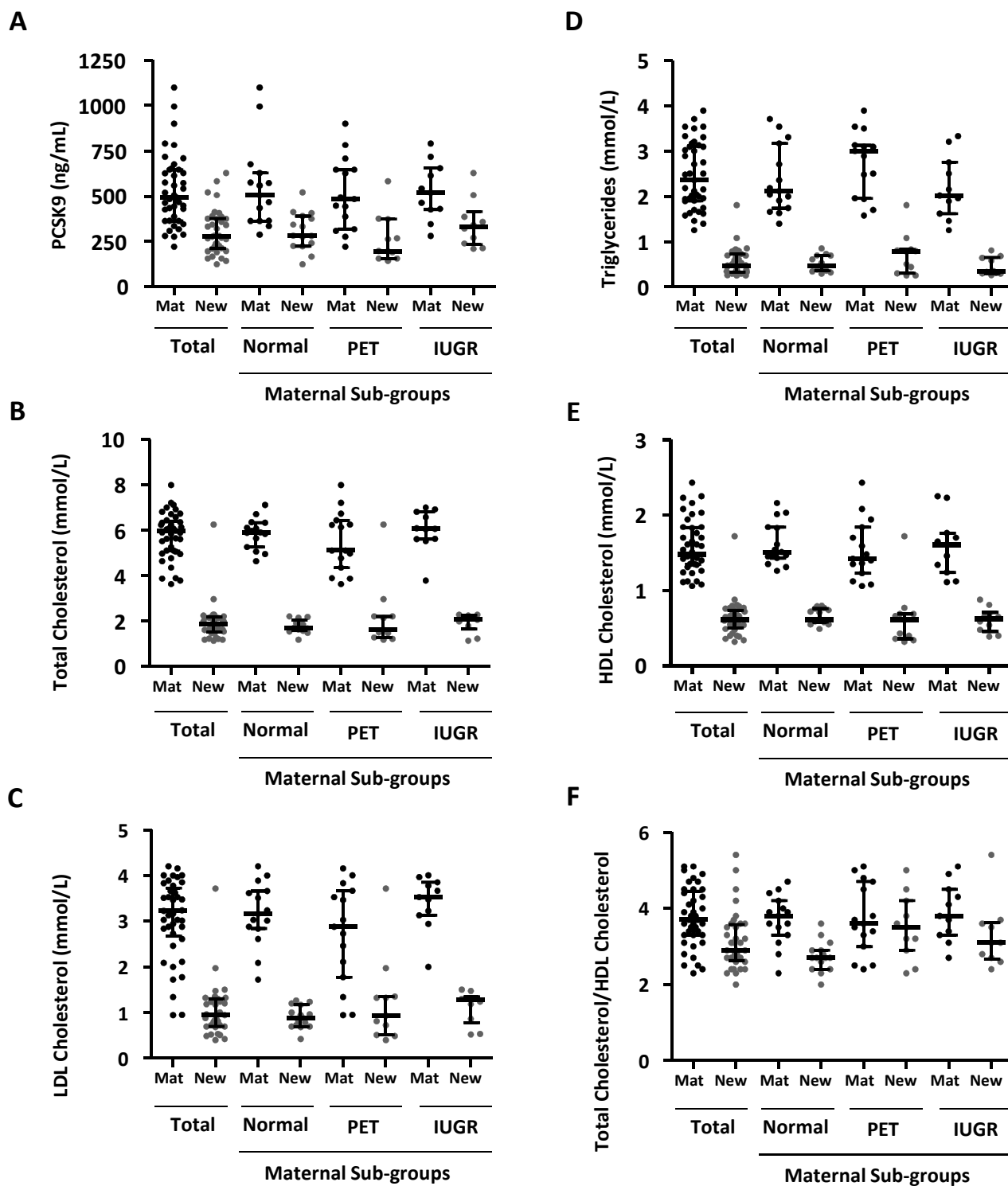

Supplemental Figure 1

Supplement: Supplementary file 1 — Figure S1: Scatter plot distributions of serum PCSK9 and lipids for the Maternal subgroups in comparison to the overall Maternal distribution, and for the Maternal subgroups (in both mother and newborn) to the overall Maternal and Newborn distributions of PCSK9 and lipids. Maternal and Newborn samples were subdivided as Normal, PET (pre-eclampsia) and IUGR (intrauterine growth restriction). Pregnancy was defined as Normal if associated with age appropriate fetal weights, in the absence of maternal disease. PET was defined as having maternal blood pressure of >140/90 and proteinuria >300 mg of protein in a 24 hr urine collection, or 2+ protein on urine dipstick. IUGR was defined as an estimated fetal weight below the 5th percentile for gestational age by ultrasound. Dots represent individual subjects. Bars indicate median ± interquartile range. No subgroup comparisons were carried out because subgrouped data were not significantly powered. [file 341632.f1.pdf]
